# Primary Prevention Through Prophylactic Mastectomy and Breast Reconstruction: An Exploratory Study on Patient Satisfaction and Quality of Life

**DOI:** 10.3390/jcm14228093

**Published:** 2025-11-15

**Authors:** Delia Nicoara, Ioan Constantin Pop, Maximilian Vlad Muntean, Radu Alexandru Ilies, Patriciu Andrei Achimas-Cadariu

**Affiliations:** 1 Faculty of Medicine, “Iuliu Hațieganu” University of Medicine and Pharmacy, 400012 Cluj-Napoca, Romania; drdelianicoara@gmail.com (D.N.); ilies.radu.alexandru@elearn.umfcluj.ro (R.A.I.); 2 Department of Quality Management, “Prof. Dr. I. Chiricuță” Institute of Oncology, 400015 Cluj-Napoca, Romania; 3 Department of Plastic Surgery, “Prof. Dr. I. Chiricuță” Institute of Oncology, 400015 Cluj-Napoca, Romania; 4 Department of Plastic and Reconstructive Surgery, “Iuliu Hațieganu” University of Medicine and Pharmacy, 400012 Cluj-Napoca, Romania; 5 Department of Surgical Oncology, “Prof. Dr. I. Chiricuță” Institute of Oncology, 400015 Cluj-Napoca, Romania; pachimas@umfcluj.ro; 6 Department of Surgical Oncology and Gynecologic Oncology, “Iuliu Hațieganu” University of Medicine and Pharmacy, 400012 Cluj-Napoca, Romania

**Keywords:** nipple-sparing mastectomy, implant-based reconstruction, quality of life, patient satisfaction, BREAST-Q, prophylactic surgery

## Abstract

**Background/Objectives**: Women who have genetic predisposition to breast cancer often opt for risk-reducing mastectomy with immediate reconstruction. Evaluating their satisfaction and quality of life is essential for guiding shared decision-making. **Methods**: This exploratory study assessed quality-of-life outcomes in two cohorts of patients undergoing bilateral prophylactic nipple-sparing mastectomy with immediate prepectoral implant-based reconstruction. Only patients without postoperative complications (necrosis, infection) were included. Each patient completed the BREAST-Q questionnaire both preoperatively (1–2 days before surgery) and postoperatively. **Results**: Postoperative BREAST-Q scores demonstrated significant improvement, with self-confidence increasing from 40.75 to 44.33, satisfaction with breast size and appearance from 50.42 to 58.50, and general esthetic/functional satisfaction from 26.92 to 33.17 (all *p* < 0.01). In contrast, physical comfort decreased from 48.00 to 32.42 (*p* < 0.001). Preoperative responses may have been influenced by anticipatory stress related to the imminent surgery and concern regarding the breast area to be operated. In contrast, postoperative results reflect psychological relief and satisfaction following a successful surgery, with no complications. **Conclusions**: Nipple-sparing mastectomy with immediate prepectoral reconstruction is associated with high patient-reported satisfaction and perceived improvements in quality of life, particularly regarding body image and emotional well-being. However, functional limitations such as reduced physical comfort should also be acknowledged. These findings further support evidence-based recommendations for prophylactic surgery in high-risk patients.

## 1. Introduction

Breast cancer (BC) remains a major cause of morbidity and mortality in oncology due to a complex interplay of risk factors, environmental influences, and genetic determinants that collectively contribute to disease development [1]. According to the data provided by GLOBOCAN in 2020 and 2022, the number of breast cancers has increased, with approximately 2.3 million new cases (11.6% of all cancers) and 665,684 deaths (6.9% of all cancer deaths) annually, making it the leading cause of cancer-related mortality among women [2,3].

Hereditary breast cancers are responsible for about 5–10% of the total number of breast cancers, with an inherited defect from one of the parents with a dominant model in half of these cases, respectively, de novo mutations in the other half, patients lacking any significant family history [4]. Germline mutations with the classical example of BRCA genes are commonly related to a high risk of developing BC. However, non-BRCA mutations (for instance, TP53—Li-Fraumeni syndrome; STK11—Peutz–Jeghers syndrome; PTEN—Cowden syndrome; and PALB2) are also associated with this pathology and, additionally, contribute to the apparition of other cancers [5].

Functionally, BRCA genes play an important role in the repairing process of double-strand DNA breaks (which may appear naturally or to be induced by radiation), being included in the class of tumoral suppressor genes. Their aberrant activity leads to the accumulation of mutations and abnormal cell divisions, resulting in genetic instability and enabling cells to acquire malignant potential, ultimately leading to the formation of neoplasms [6].

The BRCA 1 gene is involved in various processes, including the regulation of the cell cycle, transcription, and even remodeling of chromatin. The BRCA2 gene regulates the activity of RAD51, which also takes part in the repairing of the DNA [6]. Both BRCA mutations are associated with increased risk of developing BC, most of them representing non-special-type ductal carcinomas [7]. From a molecular point of view, BRCA mutations commonly lead to triple negative breast cancers (absence of estrogen receptors, progesterone receptors and HER2 expression [8]. The aim of the treatment in the group of patients with BRCA mutation-associated breast cancer is to prevent recurrence of the initial cancer and the apparition of second breast cancers and ovarian cancers [6].

By the age of 70 years, any pathogenic variant of BRCA (either BRCA 1 or 2 mutations) increase the risk of BC by 65% (44–78%) and 45% (31–56%), respectively, by 39% (18–54%) and 11% (2.4–19%) for ovarian cancer development. Other malignancies are claimed to be caused by BRCA mutations, including fallopian tube, endometrial, prostate, pancreatic, and colorectal cancer, as well as melanoma. The clinicopathological characteristics of BRCA mutations are different depending on the gene that is affected by the mutation (BRCA 1 and BRCA 2). From a histopathological point of view, about 75% of BRCA 1 pathogenic variants are linked to invasive ductal carcinoma, and 10% of cases are associated with atypical medullary cancers. As for BRCA2, lobular or ductal cancer with lobular types is found more frequently. Based on the receptor subtypes (the molecular subtypes of BC), triple-negative breast cancer occurs in 10–15% of sporadic BC and 66–100% of BRCA 1 pathogenic variants. On the other hand, only 14–35% of TBNC cases are carried by BRCA 2 mutations [9].

Nowadays, risk assessment plays an important role: all women require assessment of BC history starting from the age of 18 years and should be counseled on breast awareness and certain modifications in their lifestyle such as physical activity, weight loss, reduced alcohol intake, and smoking cessation. Women that are part of the above-average risk of breast cancer group are those with either personal or family history of BC, any known positive genetic mutation, a history of radiation in the chest before the age of 30, a history of lesions with a high risk, and with dense breast tissue. To determine which patients have a high risk of developing BC, risk calculators are available and encouraged, as these patients will benefit from annual breast screening using MRI and risk-reducing medications. What is more, high-risk patients should present to specialists in breast cancer prevention, including oncologists and genetic counselors who can further guide them and manage screening and prevention [10].

Women with a high risk of BC development represent a wide group split into subgroups, including women with and without high-risk germline mutations. It is vital that these cases are managed in a multidisciplinary manner, ensuring the optimal strategy for prevention. Selective estrogen modulators (SERMs) with the example of tamoxifen and aromatase inhibitors (AIs: anastrozole, letrozole, exemestane) proved to reduce the risk of BC development [11]. The primary preventive effect of tamoxifen for BRCA1/2 patients was tested in an analysis conducted by the National Surgical Adjuvant Breast and Bowel Project. The risk ratios to develop BC in these patients who underwent chemoprevention with tamoxifen was 1.67% (0.32–10.7%) and 0.38% (0.06–1.56%). According to these results, tamoxifen has a very limited effect with high variances depending on the case, with an overall better effect in the BRCA2 group [12].

To a greater extent, other methods of prevention, including surgery, can be applied, as bilateral risk-reducing mastectomy (BRRM) has the aim to reduce the risk of BC development in the group of BRCA mutation carriers. Bilateral risk-reducing salpingo-oophorectomy (BRRSO) is even more invasive, and it can be used to decrease the risk in BRCA2 carriers, as they can potentially develop breast cancers and ovarian neoplasms with a higher risk [11].

BRCA mutation carriers can be optimally managed in terms of therapy using a multidisciplinary approach. Consulting the guidelines is of the utmost importance, as each prophylactic measure has its own advantages and disadvantages. According to them, a risk-reducing bilateral salpingo-oophorectomy ought to be performed between 35 and 40 years in the category of patients positive for BRCA1 mutation, respectively, between 40 and 45 years for BRCA2 mutation. It is also recommended to perform annual screening for breast cancer starting from 30 years old, using mammography or breast MRI. Another recommendation is to perform BRRM with nipple-sparing mastectomy and immediate breast reconstruction for patients with BRCA pathological variants between 30 and 40 years. Because these procedures can cause morbidity and may be traumatizing for patients, psychological support is essential and must be integrated into patient care [13].

The optimal surgical management of patients with BRCA pathogenic variants remains a debatable subject. Prophylactic mastectomies can be beneficial for these patients from an oncological point of view, but positive body image and self-consciousness can be the drawbacks of this procedure, accompanied by dissatisfaction with scars, loss of sensation in the operated breast and the possibility of feeling less sexually attractive [14]. Support groups and psychotherapy turned out to be very helpful, with significant improvements in the patients’ mental health and improved quality of life [15].

This study aims to evaluate the safety and efficacy of prophylactic mastectomy followed by immediate breast reconstruction in BRCA mutation carriers compared to their preoperative status. By using standardized tools such as the BREAST-Q, we will assess patients’ quality of life, physical and emotional well-being, and satisfaction with body image both before and after surgery.

The central objective is to demonstrate that, for many patients, quality of life and satisfaction after prophylactic surgery are acceptable, particularly regarding body image and emotional well-being. By objectively documenting these outcomes, this study seeks to contribute significantly to preventive medicine, helping optimize the decision-making process and multidisciplinary management in high-risk patients.

Additionally, through this research, we aim to educate and encourage patients by showing that prophylactic mastectomy with reconstruction yields consistently positive results and should not be perceived with fear or hesitation.

Given the limited available evidence and the lack of comparative data, this work should be regarded as an exploratory, hypothesis-generating study rather than a confirmatory analysis.

## 2. Materials and Methods

This exploratory study performs a pre–post analysis (before and after intervention) and aims to assess patient satisfaction and quality of life following bilateral prophylactic mastectomy with immediate reconstruction. It is based on data collected from patients focusing on the aforementioned topic. All patients included in the study underwent nipple-sparing mastectomy with immediate prepectoral implant-based reconstruction. The procedures were executed between March 2024 and May 2025 at The Oncology Institute “Prof. Dr. I. Chiricuță” Cluj-Napoca. Exclusion criteria included postoperative complications (such as necrosis or infections).

The study population included two groups:

(a) Individuals at high genetic risk for developing breast cancer (e.g., BRCA mutation carriers) without a prior cancer diagnosis;
(b) Patients with a history of unilateral breast cancer who underwent genetic testing following diagnosis and were subsequently identified as carriers of pathogenic mutations (e.g., BRCA1/2). In these cases, a contralateral prophylactic mastectomy was recommended and performed simultaneously with oncologic surgery.

By analyzing both cohorts, the study seeks to provide a comprehensive evaluation of quality-of-life outcomes in women undergoing prophylactic mastectomy and immediate reconstruction, supporting evidence-based recommendations for surgical risk-reduction strategies in genetically predisposed patients.

Both patient satisfaction and quality of life were evaluated using the BREAST-Q questionnaire, which is a globally validated instrument that was designed to capture the patient’s perspective on the outcomes of breast surgery. This tool enables the assessment of multiple key domains, such as satisfaction with breast appearance, psychosocial and sexual well-being, as well as the satisfaction with the healthcare team and overall medical care.

To adapt the questionnaire to the specific context of prophylactic mastectomy with immediate reconstruction, a modified version of the BREAST-Q was used (the questionnaire is provided as Supplementary Material), omitting questions considered irrelevant for this patient population [16]. This adaptation has not been formally validated or psychometrically tested; therefore, the results should be interpreted with caution regarding internal validity. The questionnaires were completed anonymously at two different time points: preoperatively (1–2 days prior to surgery) and after at least six months following the procedure, allowing sufficient time for the esthetic outcomes and psychological impact to stabilize. Responses use 4- or 5-point Likert scales, with higher scores reflecting more favorable outcomes (e.g., greater satisfaction, less discomfort). For bilateral reconstruction, patients reported on the breast with lower satisfaction.

The collected data were analyzed comparatively, assessing a few differences between preoperative and postoperative responses to enable the evaluation of changes in satisfaction and quality of life. Additionally, potential factors influencing postoperative satisfaction levels were explored, aiming to identify the variables associated with higher or lower outcome scores.

To interpret the scores obtained from our modified version of the BREAST-Q questionnaire, we adopted a straightforward approach. Although some published methods suggest using complex equivalencies, interval-based categorizations, or computing total scores followed by arithmetic means, we opted for a simple summation of individual item scores for each domain. This approach allowed us to directly compare pre- and postoperative outcomes, while maintaining transparency and clarity in the analysis. Subsequent statistical evaluations were performed using basic descriptive and inferential methods, reflecting a pragmatic strategy to interpret patient-reported outcomes without overcomplicating the scoring process.

The BREAST-Q includes both positively and negatively worded items. For negatively worded items (e.g., Q3, assessing breast-related discomfort symptoms), a lower postoperative score indicates symptom improvement. For maximum clarity, the modified BREAST-Q questionnaire used in this study is provided as Supplementary Material.

In order to perform a structured analysis, BREAST-Q items were grouped into three thematic domains: esthetic outcome, functional outcome, and perception of medical care. Esthetic outcome included questions related to self-image, confidence, body perception, and satisfaction with the visual aspect of the reconstructed breast (e.g., Q1, Q2, Q4, Q5, Q7, Q10). Functional outcome encompassed physical comfort, emotional capacity, and perceived normality (e.g., Q3, Q5, Q8, Q9, Q10), acknowledging the impact of surgery on both physical and psychological well-being. Items related to satisfaction with healthcare communication and surgical planning (Q11–Q14) were grouped under perception of medical care, as these reflect patient experience beyond surgical results. Question Q5, addressing overall breast satisfaction, and Q10, addressing general outcome perception, were considered relevant to both esthetic and functional domains. Additionally, Q6, which evaluates implant rippling, was analyzed only postoperatively, as it is not applicable in the context of the natural breast. This domain-based classification enabled targeted interpretation of changes in patient-reported outcomes across key clinical dimensions.

BRCA testing was performed at patient expense due to the absence of a national reimbursement program. Patients undergoing prophylactic mastectomy were included in the national breast reconstruction program. All reconstructions were performed in a DKG-certified breast cancer center.

The statistical analysis was carried out to assess the differences regarding the BREAST-Q scores (before and after bilateral prophylactic mastectomy with immediate reconstruction). We expressed continuous date as means with standard deviations (SD). To evaluate the statistical significance of pre- versus postoperative changes for each question from the BREAST-Q questionnaire, paired-sample *t*-tests were used for normally distributed variables, whereas for non-normally distributed variables, the Wilcoxon signed-rank test was utilized.

A *p*-value < 0.05 was considered statistically significant. The statistical analyses were realized using SPSS v26.0.

## 3. Results

A total of 48 patients completed both preoperative and postoperative BREAST-Q questionnaires following bilateral prophylactic mastectomy with immediate breast reconstruction. Their responses were analyzed across three main domains: esthetic outcome, functional outcome, and perception of medical care. Postoperative questionnaires were filled completed least 6 months following surgery.

### 3.1. Esthetic Outcomes

**Figure 1:**
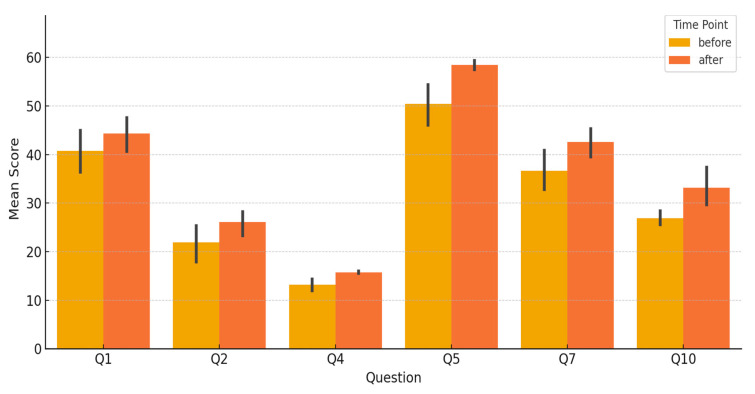
Bar Chart—Esthetic Outcome Comparison. This grouped bar chart visually compares mean preoperative and postoperative scores for each esthetic question.

Overall, patients reported a significant increase in satisfaction with esthetic outcomes postoperatively (Table 1, Figure 1). The average score for Q1 (self-confidence in social settings) increased from 40.75 preoperatively to 44.33 postoperatively. Q2 (feeling feminine in clothes) and Q4 (appearance in the mirror) also showed improvements, with respective deltas of +4.17 and +2.58. The most pronounced increase was seen in Q5 (overall satisfaction with breast size and appearance), which had an average postoperative gain of +8.08 points. Similarly, Q10 (a general esthetic satisfaction question) showed a significant increase (+6.25). Notably, concern regarding rippling (Q6) (which was not evaluated preoperatively as patients had natural breasts) was judged as satisfactory postoperatively.

Statistical analysis confirmed that all esthetic-related questions demonstrated significant differences between pre- and postoperative scores. Paired *t*-tests showed statistical significance for Q1, Q2, Q4, and Q7 (*p* < 0.01), while Wilcoxon signed-rank tests were used for Q5 and Q10 due to non-normal distributions of differences, both of which also reached significance (*p* < 0.01). 

### 3.2. Functional Outcomes

**Figure 2:**
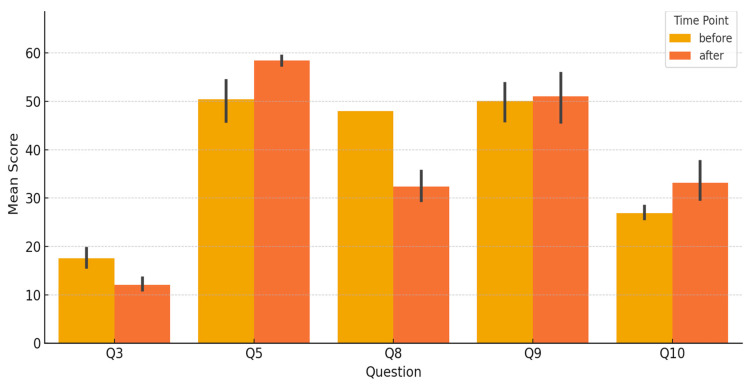
Bar Chart—Functional Outcome Comparison. This bar chart compares pre- and postoperative mean scores for functional-related questions.

Regarding the analysis of functional outcomes (Table 2, Figure 2), while Q5 and Q10 again showed improvements (mirroring the esthetic domain), other questions indicated moderate postoperative challenges. Q3 (emotional capacity) decreased by 5.50 points on average, suggesting a transient psychological burden. Q8 (physical comfort) showed the largest decline, with a delta of −15.58, indicating functional discomfort post-reconstruction. Conversely, Q9 (feeling “normal”) remained stable with minimal change (+1.00), suggesting that patients gradually adapt to their new body image.

Statistical tests revealed that the decreases in Q3 and Q8 were statistically significant (*p* < 0.001), as were the improvements in Q5 and Q10 (*p* < 0.01), further supporting the multidimensional nature of postoperative recovery.

### 3.3. Perception of Medical Care

Scores related to satisfaction with the medical team (Q11–Q14) were uniformly high and showed marginal change, reflecting a generally positive perception of care. The average score for Q11 (information from the surgeon) increased slightly from 55.58 to 58.83. Q12–Q14 remained essentially stable. Since previous studies using the BREAST-Q have reported similarly high scores in this domain, these results were not directly compared longitudinally but instead serve to affirm the consistency of high-quality surgical counseling and team communication.

## 4. Discussion

### 4.1. Interpretation of the Main Results

The findings of our study indicate that bilateral prophylactic mastectomy with immediate breast reconstruction shows higher postoperative scores suggesting perceived improvement in terms of patient-reported esthetic satisfaction, as measured by multiple validated BREAST-Q domains. The most pronounced postoperative gains were observed in satisfaction with breast size and appearance (Q5), global esthetic impression (Q10), and emotional markers such as feeling attractive and feminine (Q7, Q2). These improvements suggest that the esthetic goals of prophylactic surgery can be met or even exceeded in a high proportion of cases, particularly when patients are selected and counseled appropriately.

What is more, functional outcomes presented a more variable profile. While Q5 and Q10 also contributed positively to functional perception, patients reported declines in physical comfort (Q8) and emotional capacity (Q3) postoperatively. These findings align with the clinical experience that, despite high satisfaction with the visual result, patients may experience discomfort, tightness, or limitations in arm mobility due to reconstructive technique or implant-related factors. The observed stability in the “feeling normal” domain (Q9) suggests that, over time, patients manage to adapt to the reconstructed body image (even if transient functional limitations persist).

A separate observation is discussed regarding Q6, which evaluated postoperative rippling of the implants. Although this item was not scored preoperatively, its inclusion allows for the evaluation of implant contour irregularities. Most patients found the outcome acceptable, but the presence of visible or palpable rippling in some cases underscores the need for anticipating soft tissue coverage limitations, particularly in prepectoral reconstructions. Secondary lipofilling may serve as an effective corrective strategy in such scenarios.

Finally, satisfaction with medical care (Q11–Q14) remained high throughout the perioperative period, confirming the importance of communication, surgeon trust, and a cohesive care team in shaping patient experience.

Taken together, these results underscore the value and complexity of prophylactic mastectomy with immediate reconstruction: while esthetic gains are clearly documented and statistically supported, functional and sensory outcomes require realistic patient expectations and individualized planning.

### 4.2. Overview of the Existing Literature

Both primary prevention and early detection of hereditary BC has been widely discussed topics in the recent decades, and the availability of risk calculators allows for optimal recommendations for risk-reducing surgeries, along with optimal support via counseling. One key component of the surveillance is represented by contrast-enhanced MRIs, performed annually, fortified by biannual breast ultrasonography and mammography [17].

The attitude and acceptance rates of genetic testing among patients are important to comprehensively understand their behavioral and psychological impact in relation to the susceptibility of malignancies like breast cancer. The potential negative reaction to an unwanted result delivered by genetic testing combined with the anxiety of such a result can be very stressful for the other family members, too. Abdel-Razeq et al. analyzed the impact of testing and risk-reducing interventions towards the attitude of the patients, claiming that a lack of awareness in the view of proper preventive measures to diminish the risk of BC development is a current problem in this field, as the costs and fear of a potential unwanted result of cancer risk are the most influential causes to prevent cascade testing [18].

A main concern about the surgical treatment of BRCA1/BRCA2 mutations breast cancers is whether breast conserving surgery (BCS) combined with radiotherapy is equivalent to radical mastectomy or not. No significant difference was noticed between the survival of these two groups in one study for a 15-year survival rate (BCS: 91.7% vs. mastectomy: 92.8%, *p* = 0.85), but in BCS group a higher local recurrence was reported ipsilaterally compared to the mastectomy group (23.5% vs. 5.5%, *p* < 0.0001) [19].

The same issue is discussed in a review conducted by Vallard et al., highlighting the argument against BCT in BRCA mutation carriers if they do not undergo contralateral prophylactic mastectomy, with an increased risk of developing new disease in the contralateral breast. However, they conclude that BCT is a reasonable option for BRCA1/2 patients that are positive for mutation, due to the same local control (short term), an equivalent metastasis-free survival and an identical overall survival as in patients with sporadic BC [20].

Postoperative satisfaction after prophylactic mastectomy and breast reconstruction can be influenced by a wide range of factors, such as demographic characteristics, clinical data, and psychosocial aspects. Assessment methods that make use of the BREAST-Q questionnaire indicate that older age is correlated with higher satisfaction scores, particularly in domains like psychosocial status and sexual well-being. A prospective cohort study conducted by Shiraishi et al. aimed to evaluate satisfaction rate and health-related quality of life (after breast reconstruction, respectively, to identify the clinical factors which are associated with BREAST-Q scores at 1 and 5 years postoperatively, with a total number of 141 being involved. The results indicate that breast reconstruction performed either by using tissue expanders (or implants) or deep inferior epigastric perforator (DIEP) flaps, were significantly associated with increased “Satisfaction with Breasts” and “Psychosocial Well-being” when compared to mastectomy alone. Among the factors which were found to negatively affect “Satisfaction with Breasts” at 1 year is the increased body mass index (BMI). In addition, a positive history of psychiatric or neurological medication had an impact on “Physical Well-being.” After 5 years, reconstruction using the DIEP flap provided the best outcomes, especially in the sections “Satisfaction with Breasts” and “Psychosocial Well-being.” Bilateral procedures constituted a risk factor for lower “Psychosocial Well-being” [21].

Since there exists a multitude of measures that can be implemented for prevention, their cost-effectiveness should be analyzed, too. A variety of modifiable risk factors can be applied as a primary prevention method and are strongly encouraged: behavioral changes, reducing alcohol intake, maintaining a normal body weight, respectively, regular physical activity. All of these play their own role in reducing the risk of BC development. A review conducted by Bellanger et al. discusses the importance of cost-effectiveness in prevention programs, performing a comparison between each method. This study evidence that diet-related measures combined with physical activity as methods of primary prevention of BC are cost-effective. Public health professionals are advised to use a holistic approach in the same manner they can opt for drug therapies or reducing-risk surgical procedures, as each one of these measures has its own efficiency [22].

A multidisciplinary approach is vital when addressing the wide variety of complexities specific to prophylactic mastectomy and breast reconstruction, such as evaluating patient satisfaction and quality of life. A strong collaboration between specialists like surgeons, oncologists, psychologists, and social workers allows for a comprehensive understanding of all the medical, psychological, and social issues which influence outcomes. It has been shown that breast reconstruction is able to enhance both the body image and psychosocial well-being, even though it also carries several physical and emotional risks, which are significant [23]. Multidisciplinary medical teams have the advantage of providing balanced counseling, empowering patients with information, while addressing various concerns in relation to physical recovery, psychological adaptation, and long-term quality of life [21].

### 4.3. Limitations of the Current Study

One of the primary limitations that can be identified in this study is the relatively small sample size, which may affect the statistical power and limit the generalizability of the results to broader populations. The inclusion of patients from a single medical center further restricts the diversity of the sample. However, even if no formal sample size calculation was performed, the number of 48 patients in our cohort is acceptable taking into account the surgical intervention which was performed (prophylactic nipple-sparing mastectomy with immediate prepectoral reconstruction) in our national context.

Moreover, all participants voluntarily opted for undergoing prophylactic mastectomy with immediate reconstruction, which may cause selection bias. These individuals could possess distinct psychological profiles, risk perceptions, or levels of health awareness compared to those who declined surgery, potentially influencing the findings.

Another important limitation regarding the methodology is the absence of a separate control group. The current study followed a pre–post design (each patient served as her own control) which is widely accepted in studies assessing patient-reported outcomes after surgical interventions. However, such a design does not fully eliminate the influence of potential confounding factors such as psychological relief in the postoperative period or the aforementioned selection bias among individuals who opted for prophylactic mastectomy. Given the absence of a control group, the results of the current study should not be interpreted as evidence of causal improvement, but rather as descriptive observations of patient-reported trends.

Postoperative data were collected at a minimum of six months after surgery, a period that was considered sufficient for the stabilization of initial perceptions regarding esthetic and psychological outcomes. However, this timeframe may not be sufficient to fully capture the patient’s satisfaction or quality of life. Changes in body image, emotional adjustment, and medical complications can occur well beyond the six-month mark, and longer follow-up would be necessary to assess the durability of the perceived benefits or dissatisfaction associated with the procedure.

In addition, a major limitation is represented by the use of a modified version of the BREAST-Q, which (although conceptually based on a validated instrument) was not subjected to psychometric validation in this context. Thus, these results should be interpreted as exploratory rather than definitive.

The responses collected through the BREAST-Q questionnaire are inherently subjective and may vary significantly depending on the emotional state of the patient at the time of completion. Satisfaction and quality of life are dynamic concepts that may vary over time, making it difficult to realize an objective assessment.

Patients completed the preoperative BREAST-Q questionnaire 1–2 days prior to surgery. This timing may have influenced their psychological perception of their breasts, as the imminent surgical intervention could have amplified stress, anxiety, and concern about the affected body area. Consequently, lower preoperative scores may reflect this anticipatory emotional burden. In contrast, the postoperative assessment showed significantly improved scores, not only due to the successful esthetic and functional outcome of the prepectoral nipple-sparing mastectomy with implant-based reconstruction, but also because patients were psychologically relieved, having overcome the surgery without complications. This reduction in psychological stress may have positively influenced their perception of body image and functional recovery.

This study only included patients without postoperative complications, which allowed us to assess patient-reported outcomes under optimal surgical conditions. Consequently, our findings may not fully reflect the experience of all patients undergoing risk-reducing mastectomy with immediate implant-based reconstruction, particularly those experiencing postoperative complications.

Consequently, the variability in individual experiences may limit the consistency and reproducibility of the results, in spite of the standardized nature of the questionnaire.

## 5. Conclusions

This exploratory study indicates that bilateral prophylactic mastectomy with immediate reconstruction is linked to high patient-reported esthetic satisfaction and improved body image. While overall satisfaction is high, functional outcomes show greater variability, with some patients experiencing discomfort or reduced emotional capacity postoperatively. Consistently high ratings for medical care underscore the importance of effective communication and multidisciplinary support. These findings highlight the need for individualized surgical planning, realistic patient expectations, and, when necessary, secondary corrective procedures such as lipofilling to optimize long-term outcomes. Further controlled and larger-scale studies are required to confirm these preliminary findings and, consequently, to strengthen the evidence base for patient-reported outcomes after prophylactic mastectomy.

## Figures and Tables

**Table 1:** Esthetic Outcome (Mean ± SD per Question). This table summarizes the preoperative and postoperative BREAST-Q scores for esthetic-related questions (Q1, Q2, Q4, Q5, Q7, Q10).

Question	Item Description	Mean ± SD (Pre)	Mean ± SD (Post)
Q1	Self-confidence in social settings	40.75 ± 8.19	44.33 ± 6.64
Q2	Feeling feminine in clothes	21.92 ± 6.92	26.08 ± 4.68
Q4	Appearance in the mirror	13.17 ± 2.21	15.75 ± 0.45
Q5	Satisfaction with breast size, appearance	50.42 ± 7.74	58.50 ± 1.73
Q7	Feeling attractive	36.67 ± 7.35	42.58 ± 5.20
Q10	General esthetic/functional satisfaction	26.92 ± 2.50	33.17 ± 7.26

**Table 2:** Functional Outcome (Mean ± SD per Question). This table presents functional BREAST-Q scores (Q3, Q5, Q8, Q9, Q10), listing mean ± standard deviation values before and after surgery.

Question	Item Description	Mean ± SD (Pre)	Mean ± SD (Post)
Q3	Emotional capacity	17.58 ± 3.55	12.08 ± 2.31
Q5	Satisfaction with breast size, appearance	50.42 ± 7.74	58.50 ± 1.73
Q8	Physical comfort	48.00 ± 0.00	32.42 ± 5.71
Q9	Feeling “normal”	50.08 ± 7.08	51.00 ± 8.67
Q10	General esthetic/functional satisfaction	26.92 ± 2.50	33.17 ± 7.26

## Data Availability

Data are available upon reasonable request from the corresponding author.

## Supplementary Materials

The following supporting information can be downloaded at: https://www.mdpi.com/article/10.3390/jcm14228093/s1, The modified BREAST-Q questionnaire.
