# Supplementary material for: Primary Prevention Through Prophylactic Mastectomy and Breast Reconstruction: An Exploratory Study on Patient Satisfaction and Quality of Life
Source: J Clin Med. 2025 Nov 15;14(22):8093. doi: 10.3390/jcm14228093 (PMC12653024; doi:10.3390/jcm14228093)
Supplement: Supplementary file 1 [file jcm-14-08093-s001.zip › jcm-3853619-supplementary.pdf]

Modified BREAST-Q®

Reconstruction Module

Pre- and Postoperative Scales

English Version

Translated by a local academic, 2022.

The BREAST-Q, authored by Drs. Andrea Pusic, Anne Klassen and Stefan Cano, is the copyright of Memorial Sloan Kettering Cancer Center, The University of British Columbia, McMaster University, and Brigham and Women's Hospital (Copyright ©2017, Memorial Sloan Kettering Cancer Center). The BREAST-Q has been provided under license and must not be copied, distributed, or used in any way without the prior consent of Memorial Sloan Kettering Cancer Center.

1. With your breast area in mind, in the past week, how often have you felt:

	None of the time	A little of the time	Some of the time	Most of the time	All of the time
a. Confident in a social setting?	**1**	**2**	**3**	**4**	**5**
b. Emotionally able to do the things that you want to do?	**1**	**2**	**3**	**4**	**5**
c. Emotionally healthy?	**1**	**2**	**3**	**4**	**5**
d. Of equal worth to other women?	**1**	**2**	**3**	**4**	**5**
e. Self-confident?	**1**	**2**	**3**	**4**	**5**
f. Feminine in your clothes?	**1**	**2**	**3**	**4**	**5**
g. Accepting of your body?	**1**	**2**	**3**	**4**	**5**
h. Normal?	**1**	**2**	**3**	**4**	**5**
i. Like other women?	**1**	**2**	**3**	**4**	**5**
j. Attractive?	**1**	**2**	**3**	**4**	**5**

© 2009, 2017 Memorial Sloan-Kettering Cancer Center, Memorial Hospital for Cancer and Allied Diseases, Sloan-Kettering Institute for Cancer Research, and The University of British Columbia. All rights reserved.

2. Thinking of your sexuality, how often do you generally feel:

	None of the time	A little of the time	Some of the time	Most of the time	All of the time
a. Sexually attractive in your clothes?	**1**	**2**	**3**	**4**	**5**
b. Comfortable/at ease during sexual activity?	**1**	**2**	**3**	**4**	**5**
c. Confident sexually?	**1**	**2**	**3**	**4**	**5**
d. Satisfied with your sex-life?	**1**	**2**	**3**	**4**	**5**
e. Confident sexually about how your breast area looks when unclothed?	**1**	**2**	**3**	**4**	**5**
f. Sexually attractive when unclothed?	**1**	**2**	**3**	**4**	**5**

© 2009, 2017 Memorial Sloan-Kettering Cancer Center, Memorial Hospital for Cancer and Allied Diseases, Sloan-Kettering Institute for Cancer Research, and The University of British Columbia. All rights reserved.

3. In the past week, <u>how often</u> have you experienced:

	None of the time	Some of the time	All of the time
a. Pain in the muscles of your chest?	**1**	**2**	**3**
b. Difficulty lifting or moving your arms?	**1**	**2**	**3**
c. Difficulty sleeping because of discomfort in your breast area?	**1**	**2**	**3**
d. Tightness in your breast area?	**1**	**2**	**3**
e. Pulling in your breast area?	**1**	**2**	**3**
f. Nagging feeling in your breast area?	**1**	**2**	**3**
g. Tenderness in your breast area?	**1**	**2**	**3**
h. Sharp pains in your breast area?	**1**	**2**	**3**
i. Aching feeling in your breast area?	**1**	**2**	**3**
j. Throbbing feeling in your breast area?	**1**	**2**	**3**

Post-operative only

k. Swelling of the arm (lymphedema) on the side(s) that you had your breast surgery?	**1**	**2**	**3**

© 2009, 2017 Memorial Sloan-Kettering Cancer Center, Memorial Hospital for Cancer and Allied Diseases, Sloan-Kettering Institute for Cancer Research, and The University of British Columbia. All rights reserved.

4. With your breast area in mind, in the past week, how <u>satisfied or dissatisfied</u> have you been with:

	Very Dissatisfied	Somewhat Dissatisfied	Somewhat Satisfied	Very Satisfied
a. How you look in the mirror <u>clothed</u>?	**1**	**2**	**3**	**4**
b. How comfortably your bras fit?	**1**	**2**	**3**	**4**
c. Being able to wear clothing that is more fitted?	**1**	**2**	**3**	**4**
d. How you look in the mirror <u>unclothed</u>?	**1**	**2**	**3**	**4**

© 2009, 2017 Memorial Sloan-Kettering Cancer Center, Memorial Hospital for Cancer and Allied Diseases, Sloan-Kettering Institute for Cancer Research, and The University of British Columbia. All rights reserved.

5. If you have had a mastectomy and reconstruction of both breasts, answer these questions thinking of the breast you are least satisfied with. With your breasts in mind, in the past week, how satisfied or dissatisfied have you been with:

	Very Dissatisfied	Somewhat Dissatisfied	Somewhat Satisfied	Very Satisfied
a. How you look in the mirror clothed?	1	2	3	4
b. The shape of your reconstructed breast(s) when you are wearing a bra?	1	2	3	4
c. How normal you feel in your clothes?	1	2	3	4
d. The size of your reconstructed breast(s)?	1	2	3	4
e. Being able to wear clothing that is more fitted?	1	2	3	4
f. How your breasts are lined up in relation to each other?	1	2	3	4
g. How comfortably your bras fit?	1	2	3	4
h. The softness of your reconstructed breast(s)?	1	2	3	4
i. How equal in size your breasts are to each other?	1	2	3	4
j. How natural your reconstructed breast(s) looks?	1	2	3	4
k. How naturally your reconstructed breast(s) sits/hangs?	1	2	3	4
l. How your reconstructed breast(s) feels to touch?	1	2	3	4
m. How much your reconstructed breast(s) feels like a natural part of your body?	1	2	3	4
n. How closely matched (similar) your breasts are to each other?	1	2	3	4
o. How you look in the mirror unclothed?	1	2	3	4

© 2009, 2017 Memorial Sloan-Kettering Cancer Center, Memorial Hospital for Cancer and Allied Diseases, Sloan-Kettering Institute for Cancer Research, and The University of British Columbia. All rights reserved.

6. If you have implants in both breasts, answer these questions thinking of the breast you are least satisfied

with. In the past week, how satisfied or dissatisfied have you been with:

	Very Dissatisfied	Somewhat Dissatisfied	Somewhat Satisfied	Very Satisfied
a. The amount of rippling (wrinkling) of your implant(s) that you can see?	1	2	3	4
b. The amount of rippling (wrinkling) of your implant(s) that you can feel?	1	2	3	4

© 2009, 2017 Memorial Sloan-Kettering Cancer Center, Memorial Hospital for Cancer and Allied Diseases, Sloan-Kettering Institute for Cancer Research, and The University of British Columbia. All rights reserved.

Please answer these questions if you have had breast reconstruction using an implant.

NOTE: If you have breast implants on both sides, answer each question thinking about the side that bothers you the most.

7. With your breast in mind, in the PAST WEEK, how bothered have you been with:

	Extremely bothered	Moderately bothered	A little bothered	Not at all bothered
a. How your upper breast area looks when your arm is relaxed?	1	2	3	4
b. How your upper breast area looks when you do everyday activities (eg, get dressed)?	1	2	3	4
c. People noticing how your upper breast area looks?	1	2	3	4
d. How your upper breast area looks when you do physical activities that use your arm (eg, swim or golf)?	1	2	3	4
e. How your upper breast area looks when you raise your arm?	1	2	3	4
f. Any change in the shape of your breast when you move your arm?	1	2	3	4
g. Having to dress in a way to hide your upper breast area?	1	2	3	4
h. Your breast implant moving in a way that looks abnormal?	1	2	3	4
i. Not being able to wear certain clothes because of how your upper breast area looks?	1	2	3	4
j. How the skin of your upper breast area looks when you raise your arm (eg, dimpling or grooves)?	1	2	3	4

k. How your upper breast area looks when you lift something heavy?	**1**	**2**	**3**	**4**
l. How much your breast appearance <u>changes</u> when you flex your chest muscles?	**1**	**2**	**3**	**4**

© 2021 Memorial Sloan-Kettering Cancer Center, Memorial Hospital for Cancer and Allied Diseases, Sloan-Kettering Institute for Cancer Research, McMaster University, and Brigham and Women's Hospital. All rights reserved.

8. We would like to know how much breast sensation you have. Please answer thinking of the PAST WEEK.

<u>NOTE</u>: If you have less sensation on one side, please answer each question thinking about the side with less feeling.

	**I have <u>no</u> feeling**	**I have a <u>little</u> feeling**	**I have <u>some</u> feeling**	**I have a <u>lot</u> of feeling**	**I have <u>complete</u> feeling**
a. How much feeling do you have if you massage your breast area deeply?	**1**	**2**	**3**	**4**	**5**
b. How much feeling do you have if you press your breast area firmly?	**1**	**2**	**3**	**4**	**5**
c. How much feeling do you have if you lay on your stomach in bed?	**1**	**2**	**3**	**4**	**5**
d. How much feeling do you have if you accidently bump your breast area?	**1**	**2**	**3**	**4**	**5**
e. How much feeling do you have if you hug someone?	**1**	**2**	**3**	**4**	**5**
f. How much feeling do you have if you touch your breast area lightly?	**1**	**2**	**3**	**4**	**5**
g. How much feeling do you have if you take a shower (eg, feel the water)?	**1**	**2**	**3**	**4**	**5**
h. How much feeling do you have if you touch your breast area through clothing?	**1**	**2**	**3**	**4**	**5**
i. How much feeling do you have if your breast area is touched sexually?	**1**	**2**	**3**	**4**	**5**

© 2021 Memorial Sloan-Kettering Cancer Center, Memorial Hospital for Cancer and Allied Diseases, Sloan-Kettering Institute for Cancer Research, McMaster University, and Brigham and Women's Hospital. All rights reserved.

9. We would like to know about symptoms you may feel in your breast area.

<u>NOTE</u>: If you have more symptoms on one side, please answer each question thinking about the side that

has more symptoms.

How much do you experience these breast symptoms? Please answer thinking of the PAST WEEK.

	**A lot**	**Quite a bit**	**A little**	**Not at all**
a. Stinging pain in your breast area?	**1**	**2**	**3**	**4**
b. Throbbing feeling in your breast area?	**1**	**2**	**3**	**4**
c. Tingling in your breast area (ie, pins and needles feeling)?	**1**	**2**	**3**	**4**
d. Burning sensation in your breast area?	**1**	**2**	**3**	**4**
e. Breast area feeling swollen?	**1**	**2**	**3**	**4**
f. Sharp pain in your breast area?	**1**	**2**	**3**	**4**
g. Spasms in your breast area (ie, twitches)?	**1**	**2**	**3**	**4**
h. Breast area feeling tender?	**1**	***2***	***3***	**4**
i. Pressure in your breast area?	**1**	**2**	**3**	**4**
j. A feeling of fullness in your breast area?	**1**	**2**	**3**	**4**
k. Aching feeling in your breast area?	**1**	**2**	**3**	**4**
l. Heavy feeling in your breast area?	**1**	**2**	**3**	**4**
m. Pulling sensation in your breast area?	**1**	**2**	**3**	**4**
n. Tightness in your breast area?	**1**	**2**	**3**	**4**
o. Breast area feeling unnatural?	**1**	**2**	**3**	**4**

© 2021 Memorial Sloan-Kettering Cancer Center, Memorial Hospital for Cancer and Allied Diseases, Sloan-Kettering Institute for Cancer Research, McMaster University, and Brigham and Women's Hospital. All rights reserved.

10. Breast surgery can reduce breast sensation. We would like to know how breast sensation affects your quality of life. Please answer thinking of the PAST WEEK. How much has loss of sensation in your breast area:

	**Very much**	**Quite a bit**	**A little bit**	**Not at all**
a. …affected your ability to enjoy life?	**1**	**2**	**3**	**4**
b. …affected your self-confidence?	**1**	**2**	**3**	**4**

c. …affected your ability to move on from cancer?	**1**	**2**	**3**	**4**
d. …affected your comfort during physical activity (eg, run, swim)?	**1**	**2**	**3**	**4**
e. …affected how you feel about your body?	**1**	**2**	**3**	**4**
f. …affected the choice of clothes you wear (eg, low cut shirt or dress)?	**1**	**2**	**3**	**4**
g. …affected how comfortable bras feel?	**1**	**2**	**3**	**4**
h. …affected your sexual life?	**1**	**2**	**3**	**4**

© 2021 Memorial Sloan-Kettering Cancer Center, Memorial Hospital for Cancer and Allied Diseases, Sloan-Kettering Institute for Cancer Research, McMaster University, and Brigham and Women's Hospital. All rights reserved.

11. How satisfied or dissatisfied were you with the information you received from your surgeon about:

	**Very Dissatisfied**	**Somewhat Dissatisfied**	**Somewhat Satisfied**	**Very Satisfied**
a. How the breast reconstruction surgery was to be done?	**1**	**2**	**3**	**4**
b. Healing and recovery time?	**1**	**2**	**3**	**4**
c. Possible complications?	**1**	**2**	**3**	**4**
d. The options you were given regarding types of breast reconstruction?	**1**	**2**	**3**	**4**
e. The options you were given regarding timing of your breast reconstruction (i.e., same time as your mastectomy versus later)?	**1**	**2**	**3**	**4**
f. The pros and cons of the timing of your breast reconstruction?	**1**	**2**	**3**	**4**
g. How long the process of breast reconstruction would take from start to finish?	**1**	**2**	**3**	**4**
h. What size you could expect your breasts to be after reconstructive surgery?	**1**	**2**	**3**	**4**
i. How much pain to expect during recovery?	**1**	**2**	**3**	**4**
j. What you could expect your breasts to look like after surgery?	**1**	**2**	**3**	**4**
k. How long after reconstruction surgery it would take to feel like yourself/feel normal again?	**1**	**2**	**3**	**4**
l. How the surgery could affect future breast cancer screening (e.g., mammogram, self-examinations)?	**1**	**2**	**3**	**4**

m. Lack of sensation in your reconstructed breast(s) and nipple(s)?	**1**	**2**	**3**	**4**
n. What other women experience with their breast reconstruction surgery?	**1**	**2**	**3**	**4**
o. What the scars would look like?	**1**	**2**	**3**	**4**

© 2009, 2017 Memorial Sloan-Kettering Cancer Center, Memorial Hospital for Cancer and Allied Diseases, Sloan-Kettering Institute for Cancer Research, and The University of British Columbia. All rights reserved.

12. These questions ask about your breast cancer surgeon. Did you feel that he/she:

	**Definitely Disagree**	**Somewhat Disagree**	**Somewhat Agree**	**Definitely Agree**
a. Was professional?	**1**	**2**	**3**	**4**
b. Gave you confidence?	**1**	**2**	**3**	**4**
c. Involved you in the decision-making process?	**1**	**2**	**3**	**4**
d. Was reassuring?	**1**	**2**	**3**	**4**
e. Answered all your questions?	**1**	**2**	**3**	**4**
f. Made you feel comfortable?	**1**	**2**	**3**	**4**
g. Was thorough?	**1**	**2**	**3**	**4**
h. Was easy to talk to?	**1**	**2**	**3**	**4**
i. Understood what you wanted?	**1**	**2**	**3**	**4**
j. Was sensitive?	**1**	**2**	**3**	**4**
k. Made time for your concerns?	**1**	**2**	**3**	**4**
l. Was available when you had concerns?	**1**	**2**	**3**	**4**

© 2009, 2017 Memorial Sloan-Kettering Cancer Center, Memorial Hospital for Cancer and Allied Diseases, Sloan-Kettering Institute for Cancer Research, and The University of British Columbia. All rights reserved.

13. These questions ask about members of the medical team other than the surgeon. Did you feel that they:

	**Definitely Disagree**	**Somewhat Disagree**	**Somewhat Agree**	**Definitely Agree**
a. Were professional?	**1**	**2**	**3**	**4**
b. Treated you with respect?	**1**	**2**	**3**	**4**

c. Were knowledgeable?	**1**	**2**	**3**	**4**
d. Were friendly and kind?	**1**	**2**	**3**	**4**
e. Made you feel comfortable?	**1**	**2**	**3**	**4**
f. Were thorough?	**1**	**2**	**3**	**4**
g. Made time for your concerns?	**1**	**2**	**3**	**4**

© 2009, 2017 Memorial Sloan-Kettering Cancer Center, Memorial Hospital for Cancer and Allied Diseases, Sloan-Kettering Institute for Cancer Research, and The University of British Columbia. All rights reserved.

14. These questions ask about members of the office staff (e.g., secretaries). Did you feel that they:

	**Definitely Disagree**	**Somewhat Disagree**	**Somewhat Agree**	**Definitely Agree**
a. Were professional?	**1**	**2**	**3**	**4**
b. Treated you with respect?	**1**	**2**	**3**	**4**
c. Were knowledgeable?	**1**	**2**	**3**	**4**
d. Were friendly and kind?	**1**	**2**	**3**	**4**
e. Made you feel comfortable?	**1**	**2**	**3**	**4**
f. Were thorough?	**1**	**2**	**3**	**4**
g. Made time for your concerns?	**1**	**2**	**3**	**4**

© 2009, 2017 Memorial Sloan-Kettering Cancer Center, Memorial Hospital for Cancer and Allied Diseases, Sloan-Kettering Institute for Cancer Research, and The University of British Columbia. All rights reserved.
